# Neurodevelopmental Impairments in Adult Psychosomatic Patients

**DOI:** 10.3390/jcm13185566

**Published:** 2024-09-19

**Authors:** Nils Christensen, Michael Linden, Beate Muschalla

**Affiliations:** 1 Department of Psychotherapy and Diagnostics, Technische Universität Braunschweig, 38106 Braunschweig, Germany; 2 Department of Psychosomatic Medicine, Research Group Psychosomatic Rehabilitation, Charité University Medicine Berlin, 12200 Berlin, Germany

**Keywords:** minimal cerebral dysfunction, partial performance disorders, capacity impairments, neurodevelopmental problems, neuropsychological dysfunctions

## Abstract

**Background/Objectives**: Neuropsychological, neurodevelopmental, or minimal cerebral dysfunctions (MCD) can be found in many patients with mental disorders. They can be masked by other symptoms, impair the course of the illness, and impair work and social participation. Despite a long history of research, there is still a lack of data on the spectrum, prevalence, and consequences of these dysfunctions in patients with chronic illness. In this study, we compared patients with and without a history of neurocognitive problems in childhood for present neuropsychological dysfunctions. **Methods**: A convenience sample of 1453 psychosomatic inpatients completed the MCD scale, assessing neurodevelopmental issues in childhood and current neuropsychological dysfunctions. Additional assessments were the Attention Deficit Hyperactivity Self Rating Scale (ADHS-SB) and the Symptom Checklist 90 (SCL-90). **Results**: Significant early neurodevelopmental problems were reported by 8.87% of the patients. This group also reported a significantly higher rate of MCD symptoms and general psychosomatic symptoms (SCL-90) as compared with other patients. **Conclusions**: There is a notable prevalence of neuropsychological dysfunctions in psychosomatic patients in general, and especially in those with early neurodevelopmental problems. To adequately address specific potentially participation-relevant impairments, a broader diagnostic approach is necessary, including exploration of MCD history and present neuropsychological dysfunctions.

## 1. Introduction

Neuropsychological dysfunctions can be found in many mental disorders. These are deficits in memory, attention, executive functions, and perception [1,2,3] with a wide range of variations between and within specific disorder categories [4]. For patients with schizophrenia spectrum disorders deficits in motor function, working memory, executive functions, and processing speed have been reported [5]. Patients with addictive disorders often show deficits in executive control, working memory, and decision-making [6]. Affective disorders come along with deficits in executive functions and verbal memory [7]. In anxiety patients, impairments in executive functions, verbal memory, short-term memory, and attention deficits have been found [8,9]. Patients with schizotypal personality disorder have deficits in working memory [10,11]. In borderline patients, attention deficits and emotional processing deficits are commonly found [12].

Neuropsychological or minimal cerebral dysfunctions (MCD) are found in almost all cases of attention deficit hyperactivity disorder (ADHD) and autism spectrum disorders [13,14,15,16,17]. These disorders do not only exhibit the core symptoms like attention deficits and hyperactivity or social withdrawal but also a broad spectrum of additional neuropsychological performance deficits as can already be taken from established ADHD scales, which all list many additional dysfunctions [18,19]. Such problems are not found in all patients with respective diagnoses but are only facultative symptoms and impairments. MCDs nevertheless have a negative impact on the course of illness, the suffering of patients, and most importantly their participation in life.

The causes of MCDs can be manyfold and can often not be identified in individual patients [20,21,22]. There is some agreement that they often stem from minor organic brain damage or structural peculiarities of the brain. Another term for these problems, therefore, has been minimal brain damage (MBD) [23], because the spectrum of symptoms resembles the dementia syndrome, like deficits in memory and learning, emotional control, activity and drive, executive functions, and formal thought disorders. There is evidence that genetic factors [24], brain injury during pregnancy and birth [23,25,26], traumatic brain injury in childhood [27], and meningitis [28] can lead to (neuro)psychological developmental disorders which in many cases persist into adulthood [29]. Similarly, primordial symptomatology also shows associations with neuropsychological impairments in childhood and adulthood. This could be shown, among others, for infantile enuresis [30], nail biting [31], dyslexia [32], math problems [33], motor impairments [34], and hyperarousal [35]. The assumption that deficits simply disappear in adulthood is considered highly unlikely [36]. Irrespective of the causation of MCDs, there is evidence that early cerebral damage and neuropsychological impairment can predict mental disorders such as schizophrenia [37], anxiety disorders [38], depression [39], or personality disorders [40]. Studies have suggested that neuropsychological dysfunctions are more reliable predictors of stress, functioning, and treatment outcomes than disorder-related symptoms [41,42,43,44]. Furthermore, several investigations indicate that cognitive remediation therapy (CRT) can significantly improve neuropsychological deficits and have a meaningful impact on patients’ functioning across different diagnostic groups, independent of the primary diagnosis [45,46,47].

The literature strikingly underscores that neurodevelopmental disorders (NDDs) and other mental illnesses are frequently associated. Additionally, it indicates that neuropsychological impairments often serve as better predictors of illness progression and treatment outcomes compared to disorder-specific symptoms. However, few studies systematically investigate the full spectrum of neurodevelopmental problems in childhood and their related neuropsychological impairments and psychiatric symptom patterns in adulthood.

The diversity and clinical significance of neurodevelopmental impairments have often been obscured in both scientific and clinical contexts by a predominant focus on conditions such as ADHD. This narrow focus has concentrated interest on a limited set of symptoms, leaving many aspects underexplored. Moreover, many studies tend to investigate only isolated and specific neurodevelopmental disorders.

In this context, a broader term, such as “complex neuropsychological dysfunctions” or “minimal cerebral dysfunctions (MCD)”, may be more appropriate to encompass the full range of issues. Given the variety of comorbidities, MCD requires distinct attention beyond primary diagnoses. As neurodevelopmental deficits are long-term issues that typically emerge in early childhood or even during pregnancy, early reports of related problems are crucial for recognition and diagnosis. An important research and clinical question is the extent to which such early reports are associated with MCD in later life. Data on this issue could enhance our understanding of the problem and assist in identifying patients at risk.

In the present study, we investigated a convenience sample of German patients at a psychosomatic hospital who were suffering from various psychosomatic disorders. We examined the frequency of reports of neurodevelopmental problems in childhood, their relationship with persistent neuropsychological dysfunctions in adulthood, and analyzed their correlates. The overarching goal was to explore the extent to which neurodevelopmental problems in childhood are associated with “complex neuropsychological dysfunctions” or “minimal cerebral dysfunctions (MCD)” in adulthood and to identify how affected patients may differ from other patients with mental illness across various characteristics.

## 2. Materials and Methods

### 2.1. Sample

A total of 1453 German participants were treated at a psychosomatic hospital because of various mental disorders. The routine intake assessment was in part computer based, so that the resulting data could be used for scientific analyses. The data collection took place over a two-year period, during which all patients completed the questionnaires used in this study as part of routine diagnostics after admission. Clinical diagnoses were made by the treating physicians in reference to ICD-10 criteria. Work-related information was explored by social workers according to a standardized interview checklist. Patients had a mean age of 49.14 years (SD = 9.28), with the majority being female (65.17%). The primary clinical diagnoses, as determined by their therapists, included depression (39.26%), anxiety or stress-related disorders (34.69%), personality disorders (13.93%), developmental disorders (4.84%), and schizophrenia spectrum disorders (1.49%). Additionally, 20.68% of patients presented with at least one comorbid condition.

### 2.2. Instruments

#### 2.2.1. The MCD Checklist

The MCD checklist is a validated screening instrument, which asks for concrete, circumscribed, and observable neurodevelopmental problems in childhood and at present. Thereby, two dimensions are explored: (1) the history of neuropsychological phenomena, and (2) present symptoms.

(1) The history part of the MCD checklist asks whether concrete phenomena of minor organic brain damages had happened (6 items, e.g., “I have already had a serious accident involving the head (e.g., unconsciousness).”), and “primordial symptoms” (13 items, e.g., “I know that I had problems with learning to walk.”) [19,48,49]. This part of the scale starts with the introductory statement: “If I think about my childhood, then I know or have been told…” which is followed by items like “that there were problems during my birth”. This is answered by “not true”, “may be true”, “possibly true”, “true”, “definitely true”. If participants indicate in the history part, that at least six severe or very severe problems (rating 3 or 4) have existed, then they can be classified as persons with developmental problems (DP) [48,50].
(2) The second part of the MCD checklist explores 51 present neuropsychological dysfunctions, i.e., “orientation” (3 items, e.g., “In large or winding buildings or department stores, I have no sense of where to get out.”), “memory” (5 items, e.g., “I have problems recognizing faces.”), “cognition” (7 items, e.g., “Grammar or a lot of complicated subordinate clauses get me off track.”), “vegetative lability” (7 items, e.g., “Noise always quickly becomes too much for me.”), “emotionality”(8 items, e.g., “I quickly get into different emotional states.”), “motor skills” (7 items, e.g., “When it comes to dancing, I’m rather clumsy.”), “attention” (6 items, e.g., “Careless mistakes happen to me quickly.”), and “activity”(6 items, e.g., “I am constantly active and on the move.”). Each item is rated on a Likert scale from “0 = not true at all” to “4 = very severe”. An item was considered to be clinically relevant, when the symptom severity was rated with “3 = severe” and “4 = very severe”, to exclude minor and irrelevant complaints. In clinical practice, therapists are advised to look at the spectrum of reported complaints and then make a specific examination of the problem.

A validation of the MCD checklist was carried out with a large sample of German patients (*N* = 1346) from a psychosomatic clinic [48,50]. The results demonstrated strong psychometric properties, with the scale showing satisfactory internal consistency, as indicated by a Cronbach’s alpha of 0.811 [48]. For scientific use of the MCD checklist a sum scores for all items or separately for both dimensions (history, present symptoms) can be calculated to get an idea of the overall symptom load [50]. In sum, the MCD checklist catches a broad spectrum of “neuropsychological and minimal cerebral dysfunctions”, which are (or have been in history) concrete and observable, and which are present in adulthood and come along with relevant impairments [19,48,49].

#### 2.2.2. ADHS-SB (Attention Deficit Hyperactivity Self Rating)

The ADHS-SB is a self-report questionnaire to assess ADHD symptoms in adults, with the subscales “inattention” and “hyperactivity-impulsivity” [51,52]. It is an adaptation of the Wender ADHD scale and based on the definitions in ICD-10 and DSM-IV. The items of the ADHS-SB are answered on a Likert scale from “0 = not true at all” to “3 = severe”. Based on the recommendation only items with a rating of at least “2 = moderate” or higher have been considered in our study [52]. Following the authors, the cut-off for ADHD was set at 18, which allows an ADHD diagnosis with a sensitivity of 65% and a specificity of 92% [52].

#### 2.2.3. SCL-90-R

The SCL-90 is a self-rating scale with 90 items, to assess general psychological distress [53]. There are nine subscales like aggressiveness/hostility, anxiety, depressiveness, paranoid thinking, phobic anxiety, psychoticism, somatization, insecurity in social situations, and compulsivity. Ratings are made on a five-point Likert-scale ranging from “0 = not at all” to “4 = very strong”. The global severity index (GSI) represents the average over all items.

### 2.3. Design and Statistics

All patients routinely filled in the above-described scales at the point of admission to the hospital. They were then divided into those with developmental problems (DP group) and without developmental problems (comparison group) according to the MCD checklist history section (Table 1). The cut-off was 6 positive history items out of 19. Warnke, based on the validation sample, proposed a cut-off of at least positive 4 history items for classification into the MCD group. However, considering the distribution of response frequencies observed in the validation study, we opted for a more conservative cut-off value of 6 positive history items to enhance specificity. The rating of severity had to be at least 3 or 4 to exclude minor or unclear memories.

The resulting two groups of patients were compared regarding their present MCD symptomatology, their clinical diagnoses, and other correlates. Depending on the data type relative frequencies, mean values, median, and standard deviation were compared. To assess significant differences between the two groups, chi-square tests were applied for categorical variables, while Mann–Whitney U tests were conducted for continuous variables. Data analysis was performed using R version 4.3.1 (R Foundation for Statistical Computing, Vienna, Austria).

## 3. Results

We took data from 1453 unselected patients from the hospital files. Clinical diagnoses, as given by their therapists, were depression in 39.26% of patients, anxiety, or stress disorders in 34.69%, personality disorders in 13.93%, developmental disorders in 4.84%, and schizophrenia spectrum disorders in 1.49%, with 20.68% having at least one comorbid disorder. Criteria for developmental disorders according to our criteria (DP group) were fulfilled by 8.87% of patients.

Table 1 shows the frequencies of self-reported developmental and primordial problems in childhood according to the MCD checklist. It also reports if there are significant differences in the occurrence of these problems between the DP group and the comparison group. DP patients reported birth problems in 53%, maternal mental stress during pregnancy in 46%, traumatic brain injury in 40%, failures to thrive in 31%, and distractibility (72.09%), hyperarousal (65.11%), thumb-sucking (64.34%), and hyperactivity (51.94%), learning problems (47%), dyslexia (44%), dyscalculia (44%), social problems (40%), and motor deficits (33%). In the control group, these rates were significantly and clinically relevant lower. The scope of the burdens of symptoms and differences between the DP group and controls can also be taken from the means of developmental problems.

As shown in Table 2, differences in symptom burden between the DP group and the comparison group were found across all domains of the MCD scale. But there were nevertheless also many MCD symptoms reported by the control patients. In the orientation domain, most common among DP patients were orientation problems (37.98% in DP group vs. 22.23% in the comparison group) and number reversal (20.93% vs. 3.70%). Regarding memory most frequent were word finding difficulties (50.38% vs. 23.94%) and general memory difficulties (42.63% vs. 19.03%). Cognitive and speech problems were preferably stumbling over words (52.71% vs. 22.28%) and prolixity (41.86% vs. 17.60%). Problems in vegetative regulation were noise intolerance (62.02% vs. 42.44%), tiredness (47.29% vs. 27.49%), and exhaustion (44.96% vs. 20.54%). Common emotion regulation problems were difficulties in calming down (55.81% vs. 31.95%), affect incontinence (55.04% vs. 48.87%), lack of serenity (51.94% vs. 31.95%), and affective lability (51.94% vs. 31.34%). In the motor domain, there were problems with handwriting (37.98% vs. 14.35%), dancing (35.66% vs. 21.83%), and neurological soft signs (34.88% vs. 16.62%). Regarding attention distractibility (54.26% vs. 17.67%), slip errors (58.14% vs. 21.75%), and mental leaps (51,94% vs. 24.92%) were most frequent. Activity and drive impairments were difficulties standing in line (48.84% vs. 20.85%), sit restlessness (40.31% vs. 16.77%), and hyperactivity (31.78% vs. 19.94%).

Furthermore, we calculated sum scores reflecting the number of severe problems (severity 3/4) per patient. The second part of Table 2 presents the mean values derived from these sum scores for the respective groups. The differences between groups were mostly significant and clinically relevant.

Patients with a striking neuropsychological history (DP group) were more frequently diagnosed with personality disorders (ICD-10 F6) at a rate of 20.00%, developmental disorders (ICD-10 F8) at 17.60%, and disorders with onset in childhood and adolescence (ICD-10 F8) at 2.40%, compared to the comparison group, as shown in Table 3.

Table 4 shows sociodemographic data for the DP group and the comparison group. There are significant differences which suggest that DP patients are globally more burdened as seen by the GSI score (mean = 1.62 vs. mean = 1.14), are more often single, less often married, have a lower education and more often no vocational qualification. Regarding age, no significant differences were found between the groups.

## 4. Discussion

This study has five major results. The first result is that approximately one in ten psychosomatic patients reports neurodevelopmental and primordial problems in childhood. There are events which are known to be risk factors for brain impairment like maternal somatic but also psychological stress during pregnancy [54,55], difficult birth [56], traumatic brain injury [57] and brain infections [58]. Such factors do not in all cases result in brain damage but can have a negative impact on the brain structure of the child. This can occur globally or regionally, and accordingly have different neuropsychological consequences. In some cases, the growing brain can compensate such defects. In others, they will persist or at least lead to changes in the further brain development. Considering these many options, it becomes evident that there must be a great heterogeneity of such problems regarding their type and severity. The question is whether the rate of approximately ten percent is plausible. It can be compared with other clear cut brain defects. In the general population, there are approximately 1% of severe mental handicaps [59]. Different disorders are associated with mental impairment and clearly related to early childhood or prenatal brain damage. These include cerebral palsy, the prevalence of which is estimated at 2/1000 live births [60], or epilepsy, with an estimated prevalence of almost 1% [61]. There are prevalence rates up to 11% of distinct neuropsychological abnormalities in children, like legasthenia, dyscalculia, attention deficit problems and many others [62]. Given that we did not investigate a sample of the general population but psychosomatic patients, the observed rate is quite plausible. In summary, the data show that neurodevelopmental problems should receive proper attention when taking patient history [63]. This seems to be especially true for ADHD patients. Patients in the DP group report ADHD symptoms significantly more often on the ADHD-SB scale. The results suggest that ADHD-specific symptoms may play a role in patients with neurodevelopmental problems, but not necessarily [49]. However, even in the control group, one in ten patients is conspicuous on the ADHD-SB scale. Methodological weaknesses due to the use of self-report questionnaires should be discussed at this point. It is possible that individual primordial symptoms are not remembered retrospectively or were not considered in the MCD questionnaire.

The second important result of our study shows why it is important to ask for neurodevelopmental and primordial problems in childhood. They are significant predictors of current minimal cerebral dysfunctions. Our findings support results from many other studies which show that (neuro)psychological developmental disorders in childhood correspond to MCDs in adulthood and do not simply disappear in the further course of development [15,36,64,65]. When comparing DP patients with other psychosomatic patients, who also suffer from relevant mental disorders, nevertheless significant and relevant differences can be observed.

The third important result of our study is, that it gives an idea of the manifoldness of possible MCDs, and supports similar finding from other authors [1,2,3]. They cover the full spectrum of organic brain disorders and dementia syndromes. This includes problems with orientation, memory, thinking, vegetative regulation, mood control, motor skills, attention, drive, and activity. Though in much lower rates, all symptoms are also reported by control patients. This may be explained by basic problems with self-rating scales, as individual persons may misunderstand individual items or tend to complain about everything. It may also be because such symptoms can be part of other mental disorders, which is true for mood regulation or autonomic stability, for example. The conclusion is that MCD cannot be diagnosed by looking at single symptoms or by counting symptoms but needs a prototypical diagnostic approach [66].

The fourth important result of this study is that MCDs affect patients with different clinical diagnoses. In our sample, patients with various clinical diagnoses have reported neurodevelopmental problems in childhood and symptoms of MCD in adulthood. This was particularly evident for personality disorder, disorders of psychological development, and behavioral and emotional disorders. This aligns with previous research highlighting the presence of neuropsychological deficits in developmental disorders [67], ADHD [16,18,19], and personality disorders [11]. These findings emphasize the cross-diagnosis relevance of the MCD concept in adult psychiatry.

The fifth important result is that our data confirm, that MCD has a direct impact on daily life and participation of affected persons. MCD patients show significantly lower education levels, occupational development, and family integration.

In psychotherapy, it may happen that these social problems are recognized and interpreted as causes of the present symptoms. Our data recommend changing this view and understand the symptoms as cause of social problems. Furthermore, MCD problems should be understood as mental handicap, because they are chronic, rest over the lifetime (history and present symptoms), and come along with impairment in fulfilling daily duties. In some cases, cognitive remediation therapy (CRT) may help patients to restore patients’ functioning [45,46,47]. In most cases, training and psychotherapy will not be able to change the core of the problem. Instead, treatment must focus on compensation, for example following the SOC- model (selection, optimization, compensation) under a rehabilitative perspective [68]. This means first to make a precise diagnosis of strengths and deficits. This speaks for the importance of a specific assessment of MCDs. The next step is to focus on the strength and even optimize these by training. Deficits which must be endured need compensatory strategies including the adaptation of the environment in the sense of person–environment fit.

### Limitations

When interpreting our results, methodological limitations must be considered. First, it should be noted that the ADHD questionnaire and the MCD checklist are self-rated. There is a potential for bias. It cannot be ruled out that the data are biased, for example, due to a fundamentally increased tendency of patients to complain or because primordial symptoms are no longer remembered (e.g., pregnancy difficulties of the mother). However, the MCD checklist asks for very concrete circumscribed phenomena which can be observed by the person himself and by others, and which are regularly well known and reportable (people know whether they have problems with orientation, or calculation). We have not performed additional objective assessments of selected neuropsychological phenomena. However, there is a broad variety of phenomena, and it would not be possible to assess all problems which are collected in the MCD checklist. Further clinical observations of reported present neuropsychological problems, and performance in selected neuropsychological tests, would add to the structured exploration in future studies. Furthermore, only psychosomatic inpatients were studied here. Results may be different in other clinical populations. Clinical diagnoses were made by therapists, so their validity can be questioned. Due to economic reasons in clinical basic routine diagnostic, we only have basic information on the functional level and participation restrictions in daily life. Further explorations of capacity impairments based on the International Classification of Functioning, Disability, and Health (ICF) would be useful [69].

## 5. Conclusions

Neuropsychological dysfunctions are common in psychosomatic patients. Patients with self-reported neurodevelopmental problems in their medical history show a significantly higher MCD symptom burden than comparison patients. MCD can be observed across all diagnostic groups, but patients with personality disorder, disorders of psychological development, behavioral and emotional disorders, and schizophrenia spectrum disorders tend to experience higher burdens. The inadequate consideration of MCD symptoms and mild neuropsychological impairments poses a significant issue in the clinical setting, as this patient group exhibits more severe impairments compared to other individuals with mental illness. Therefore, further research is necessary to investigate the prevalence of MCD symptoms in different populations and explore potential treatment approaches. Further research studies should also investigate whether diffuse MCD symptomatology can be objectified by neuropsychological testing and derive specific therapeutic and compensatory interventions. Therapy manuals for the treatment of neuropsychological dysfunctions are needed. Specific symptom- and function-related interventions in MCD patients might be more effective than global, disorder-related therapies.

## Figures and Tables

**Table 1:** Self-reported developmental disorders and primordial problems (DP) in childhood.

MCD Checklist: History	Total Sample (*N* = 1453)	DP Group (*N* = 129)	Comparison Group (*N* = 1324)	Significance of Difference between DP Group and Comparison Group
**Minor organic brain damages**				
Somatic stress of the mother during pregnancy	10.32%	36.43%	7.78%	χ^2^ = 104.26
				*p* < 0.001
Mental stress of the mother during pregnancy	11.70%	45.73%	8.38%	χ^2^ = 158.75
				*p* < 0.001
Difficult birth	17.48%	53.48%	13.97%	χ^2^ = 127.24
				*p* < 0.001
Meningitis	3.03%	4.65%	2.87%	χ^2^ = 1.2698
				*p* = 0.2704
Traumatic brain injury	14.04%	40.31%	11.48%	χ^2^ = 80.953
				*p* < 0.001
Failure to thrive	8.33%	31.01%	6.12%	χ^2^ = 95.39
				*p* < 0.001
**Primordial symptoms in childhood**				
Delayed motor development	3.23%	15.50%	2.04%	χ^2^ = 68.084
				*p* < 0.001
Delayed language development	4.54%	17.83%	3.25%	χ^2^ = 57.642
				*p* < 0.001
Bedwetting	6.81%	30.23%	4.53%	χ^2^ = 122.29
				*p* < 0.001
Thumb-sucking	22.16%	64.34%	18.05%	χ^2^ = 146.01
				*p* < 0.001
Tics	2.27%	8.53%	1.66%	χ^2^ = 24.962
				*p* < 0.001
Hyperarousal	15.97%	65.11%	11.17%	χ^2^ = 254.88
				*p* < 0.001
Hyperactivity	12.73%	51.94%	8.91%	χ^2^ = 195.84
				*p* < 0.001
Distractibility	16.17%	72.09%	10.73%	χ^2^ = 326.52
				*p* < 0.001
Dyslexia	12.60%	44.19%	9.52%	χ^2^ = 128.35
				*p* < 0.001
Dyscalculia	10.81%	44.19%	7.55%	χ^2^ = 163.68
				*p* < 0.001
Learning or memory disorder	9.15%	47.29%	5.44%	χ^2^ = 247.56
				*p* < 0.001
Motor deficits	11.01%	32.56%	8.91%	χ^2^ = 67.07
				*p* < 0.001
Social outsider	12.87%	40.31%	10.20%	χ^2^ = 95.06
				*p* < 0.001
**Mean history symptoms**				
Total Anamnese (19 items)	Mean = 2.05 MD = 1.00 (SD = 2.30)	Mean = 7.46 MD = 7.00 (SD = 1.62)	Mean = 1.53 MD = 1.00 (SD = 1.55)	U = 0 *p* < 0.001
Early brain damages (6 items)	Mean = 0.65 MD = 0.00 (SD = 0.96)	Mean = 2.12 MD = 2.00 (SD = 1.34)	Mean = 0.51 MD = 0.00 (SD = 0.78)	U = 26,444 *p* < 0.001
Primordial symptoms (13 items)	Mean = 1.40 MD = 1.00 (SD = 1.80)	Mean = 5.34 MD = 5.00 (SD = 1.76)	Mean = 1.02 MD = 1.00 (SD = 1.26)	U = 4926.5 *p* < 0.001

**Table 2:** Self-reported severe and very severe present MCD symptoms in patients with (DP group) and without (comparison group) developmental problems.

MCD Checklist: Present Neuropsychological Dysfunctions	Total Sample (*N* = 1453)	DP Group (*N* = 129)	Comparison Group (*N* = 1324)	Significance of Difference between DP Group and Comparison Group
**Orientation**				
Orientation in a foreign apartment	2.96%	6.98%	2.57%	χ^2^ = 7.9559 *p* < 0.05
Orientation problems in the forest	21.27%	34.88%	19.94%	χ^2^ = 15.678 *p* < 0.01
Orientation problems in foreign cities	23.54%	37.98%	22.13%	χ^2^ = 16.418 *p* < 0.001
Orientation problems in foreign builidings	16.72%	31.78%	15.26%	χ^2^ = 23.051 *p* < 0.001
Number reversal	5.23%	20.93%	3.70%	χ^2^ = 70.393 *p* < 0.001
**Memory**				
Recognize faces	9.08%	20.93%	7.93%	χ^2^ = 24.051 *p* < 0.001
Remember names	27.80%	48.06%	25.83%	χ^2^ = 28.941 *p* < 0.001
Remember numbers	15.62%	34.88%	13.75%	χ^2^ = 39.841 *p* < 0.001
Word finding difficulties	26.29%	50.38%	23.94%	χ^2^ = 42.421 *p* < 0.001
Memory difficulties	21.13%	42.63%	19.03%	χ^2^ = 39.295 *p* < 0.001
**Cognition**				
Word confusion	12.53%	31.00%	10.73%	χ^2^ = 44.134 *p* < 0.001
Stumble over words	24.98%	52.71%	22.28%	χ^2^ = 58.087 *p* < 0.001
Grammar problems	12.94%	40.31%	10.27%	χ^2^ = 94.155 *p* < 0.001
Social awkwardness	20.23%	48.06%	17.52%	χ^2^ = 67.925 *p* < 0.001
Word fluency	17.62%	37.21%	15.71%	χ^2^ = 37.433 *p* < 0.001
Prolixity	19.75%	41.86%	17.60%	χ^2^ = 43.654 *p* < 0.001
Stuttering	17.07%	40.31%	14.80%	χ^2^ = 54.026 *p* < 0.001
**Vegetative lability**				
Tiredness	29.25%	47.29%	27.49%	χ^2^ = 22.256 *p* < 0.001
Noise tolerance	44.18%	62.02%	42.44%	χ^2^ = 18.251 *p* < 0.001
Vitality	22.57%	36.43%	21.22%	χ^2^ = 15.56 *p* < 0.001
Exhaustion	22.71%	44.96%	20.54%	χ^2^ = 39.925 *p* < 0.001
Hyperexcitability	18.58%	37.21%	16.77%	χ^2^ = 32.467 *p* < 0.001
Headache	20.44%	24.81%	20.02%	χ^2^ = 1.6592 *p* = 0.2074
Hypersensitivity	23.26%	33.33%	22.28%	χ^2^ = 8.0438 *p* < 0.01
**Emotions**				
Quick temper	30.28%	49.61%	28.40%	χ^2^ = 25.056 *p* < 0.001
Lack of serenity	33.72%	51.94%	31.95%	χ^2^ = 21.014 *p* < 0.001
Affective lability	33.17%	51.94%	31.34%	χ^2^ = 22.487 *p* < 0.001
Affect incontinence	49.42%	55.04%	48.87%	χ^2^ = 1.7912 *p* = 0.2059
Undiplomatic behavior	11.22%	22.48%	10.12%	χ^2^ = 18.03 *p* < 0.001
Difficulties calming down	34.07%	55.81%	31.95%	χ^2^ = 29.806 *p* < 0.001
Affective instability	26.02%	39.53%	24.70%	χ^2^ = 13.444 *p* < 0.01
Aggressiveness	14.32%	24.03%	13.37%	χ^2^ = 10.895 *p* < 0.01
**Motor Skills**				
Handwriting	16.45%	37.98%	14.35%	χ^2^ = 47.775 *p* < 0.001
Fine motor skills (drying glasses)	4.68%	11.63%	4.00%	χ^2^ = 15.32 *p* < 0.01
Fine motor skills (steady hand)	12.66%	27.13%	11.25%	χ^2^ = 26.795 *p* < 0.001
Irregular gait	8.12%	24.81%	6.50%	χ^2^ = 52.819 *p* < 0.001
Motoric suppleness	12.66%	27.91%	11.18%	χ^2^ = 29.743 *p* < 0.001
Dancing	23.06%	35.66%	21.83%	χ^2^ = 12.676 *p* < 0.001
Neurological Soft Signs	18.24%	34.88%	16.62%	χ^2^ = 26.305 *p* < 0.001
**Attention**				
Distractibility	20.92%	54.26%	17.67%	χ^2^ = 95.12 *p* < 0.001
Slip errors	24.98%	58.14%	21.75%	χ^2^ = 83.044 *p* < 0.001
Mental leaps	27.32%	51.94%	24.92%	χ^2^ = 43.197 *p* < 0.001
Impatience in listening	20.72%	40.31%	18.81%	χ^2^ = 33.093 *p* < 0.001
Misplacing things	19.89%	37.21%	18.20%	χ^2^ = 26.651 *p* < 0.001
Patience	15.83%	30.24%	14.42%	χ^2^ = 22.043 *p* < 0.001
**Activity and drive**				
Urge to move	16.59%	28.68%	15.41%	χ^2^ = 14.971 *p* < 0.001
Sit restlessness	18.86%	40.31%	16.77%	χ^2^ = 42.579 *p* < 0.001
Increased activity level	20.99%	31.78%	19.94%	χ^2^ = 9.9415 *p* < 0.001
Difficulties standing in line	23.33%	48.84%	20.85%	χ^2^ = 51.488 *p* < 0.001
Impulsiveness in conversation	10.94%	23.26%	9.74%	χ^2^ = 22.024 *p* < 0.001
Annoying others	10.74%	20.16%	9.82%	χ^2^ = 13.104 *p* < 0.001
**Mean MCD symptoms**				
MCD symptoms without anamnesis (51 items)	Mean= 10.35 MD = 8.00 (SD = 9.27)	Mean = 19.22 MD = 18.00 (SD = 11.10)	Mean = 9.48 MD = 7.00 (SD = 8.60)	U = 40,390 *p* < 0.001
Orientation (5 items)	Mean = 0.70 MD = 0.00 (SD = 1.18)	Mean = 1.33 MD = 1.00 (SD = 1.58)	Mean = 0.64 MD = 0.00 (SD = 1.12)	U = 64,364 *p* < 0.001
Memory (5 items)	Mean = 0.99 MD = 0.00 (SD = 1.31)	Mean = 1.97 MD = 2.00 (SD = 1.60)	Mean = 0.90 MD = 0.00 (SD = 1.24)	U = 50,698 *p* < 0.001
Cognition (7 items)	Mean = 1.25 MD = 0.00 (SD = 1.95)	Mean = 2.92 MD = 2.00 (SD = 2.47)	Mean = 1.09 MD = 0.00 (SD = 1.82)	U = 46,143 *p* < 0.001
Vegetative lability (7 items)	Mean = 1.81 MD = 1.00 (SD = 1.71)	Mean = 2.86 MD = 3.00 (SD = 1.87)	Mean = 1.71 MD = 1.00 (SD = 1.67)	U = 54,674 *p* < 0.001
Emotions (8 items)	Mean = 2.32 MD = 2.00 (SD = 2.31)	Mean = 3.50 MD = 3.00 (SD = 2.49)	Mean = 2.21 MD = 1.00 (SD = 2.26)	U = 59,242 *p* < 0.001
Motor skills (7 items)	Mean = 0.96 MD = 0.00 (SD = 1.36)	Mean = 2.00 MD = 1.00 (SD = 1.90)	Mean = 0.86 MD = 0.00 (SD = 1.24)	U = 53,688 *p* < 0.001
Attention (6 items)	Mean = 1.30 MD = 0.00 (SD = 1.77)	Mean = 2.72 MD = 3.00 (SD = 2.17)	Mean = 1.16 MD = 0.00 (SD = 1.66)	U = 48,874 *p* < 0.001
Activity and drive (6 items)	Mean = 1.01 MD = 0.00 (SD = 1.49)	Mean = 1.93 MD = 1.00 (SD = 1.87)	Mean = 0.93 MD = 0.00 (SD = 1.41)	U = 56,912 *p* < 0.001

**Table 3:** Distribution of clinical diagnosis in patients with (DP group) and without (comparison group) developmental problems.

ICD-10 Primary Diagnoses	DP Group (*N* = 129)	Comparison Group (*N* = 1324)	Significance of Difference between DP Group and Comparison Group
**Diagnostic categories**			
F1: Mental and behavioral problems due to psychotropic substances	0.00%	2.79%	χ^2^ = 3.583 *p* = 0.05947
F2: Schizophrenic disorders	2.40%	1.40%	χ^2^ = 0.77696 *p* = 0.4308
F3: Affective disorders	27.20%	40.51%	χ^2^ = 8.4199 *p* < 0.05
F4: Anxiety and adjustment disorders	28.00%	34.35%	χ^2^ = 2.042 *p* = 0.1729
F5: Behavioral dysfunctions associated with somatic problems	2.40%	2.55%	χ^2^ = 0.0099532 *p* = 1.00
F6: Personality disorders	20.00%	13.31%	χ^2^ = 4.2286 *p* < 0.05
F7: Intelligence disorders	0.00%	0.90%	χ^2^ = 1.1392 *p* = 0.4158
F8: Developmental disorders	17.60%	3.53%	χ^2^ = 48.667 *p* < 0.001
F9: Behavioral and emotional disorders beginning in childhood or youth	2.40%	0.66%	χ^2^ = 4.2345 *p* = 0.07696
**One or more comorbid disorders**			
Yes	32.56%	22.72%	χ^2^ = 19.822 *p* < 0.01
No	67.44%	77.28%	

**Table 4:** Sociodemographic data of patients with (DP group) and without (comparison group) developmental problems.

		Total Sample (*N* = 1453)	DP Group (*N* = 129)	Comparison Group (*N* = 1324)	Significance of Difference between DP Group and Comparison Group
GSI-Score		Mean = 1.19 (SD = 0.66)	Mean = 1.62 (SD = 0.75)	Mean = 1.14 (SD = 0.64)	U = 53,004 *p* < 0.001
ADHD (ADHS-SB)					χ^2^ = 64.25 *p* < 0.001
	Yes	14.59%	38.76%	12.24%	
	No	85.41%	61.24%	87.76%	
Age		Mean = 49.14 (SD = 9.28)	Mean = 47.26 (SD= 10.01)	Mean = 49.32 (SD = 9.19)	U = 81,780 *p* = 0.03911
Sex					χ^2^ = 6.9056 *p* < 0.01
	Male	34.83%	45.73%	33.76%	
	Female	65.17%	54.26%	66.24%	
Relationship status					χ^2^ = 19.885 *p* < 0.01
	Single	22.00%	36.43%	20.59%	
	Married	55.03%	39.53%	56.54%	
	Divorced	19.59%	20.16%	19.53%	
	Widowed	2.90%	3.10%	2.88%	
	Other	0.48%	0.78%	0.45%	
Education					χ^2^ = 30.079 *p* < 0.001
	No school degreee	0.90%	4.65%	0.53%	
	Basic education	12.41%	19.38%	11.73%	
	Secondary education degree	56.76%	51.94%	57.23%	
	High school	29.17%	23.26%	29.75%	
	Other	0.76%	0.78%	0.76%	
Vocational qualification					χ^2^ = 18.227 *p* < 0.001
	Apprenticeship	71.31%	73.64%	71.08%	
	Master	3.86%	0.78%	4.16%	
	University degree	19.03%	13.18%	19.61%	
	No degree	4.62%	10.85%	4.01%	
	Other	1.17%	1.55%	1.14%	

## Data Availability

Data are available from the authors on request.
